# Saliva as a diagnostic specimen for detection of SARS-CoV-2 in suspected patients: a scoping review

**DOI:** 10.1186/s40249-020-00728-w

**Published:** 2020-07-22

**Authors:** Omid Fakheran, Mandana Dehghannejad, Abbasali Khademi

**Affiliations:** 1 Dental research center, Department of Periodontics, Dental Research Institute, Isfahan University of Medical sciences, Isfahan, Iran; 2 Dental Research Center, Dental Research Institute, Isfahan University of Medical sciences, Isfahan, Iran; 3 Dental Research Center, Department of Endodontics, Faculty of Dentistry, Dental Research Institute, Isfahan University of Medical Sciences, Isfahan, Iran

**Keywords:** COVID-19, SARS-CoV-2, 2019-nCoV, Saliva, PCR, Review

## Abstract

**Background:**

From the begging months of 2020 a severe acute respiratory syndrome coronavirus (SARS-CoV-2, also called 2019-nCoV) caused a devastating global outbreak. At present, the diagnosis of coronavirus disease 2019 (COVID-19) is made through a nasopharyngeal swab based on reverse transcription polymerase chain reaction (RT-PCR) technique. However, some recent studies suggested the possible role of oral fluids and saliva in the detection of SARS-CoV-2. The purpose of this scoping review is evaluating the available evidence regarding the efficacy of saliva as a diagnostic specimen in COVID-19 patients.

**Methods:**

A systematic literature review of six databases (PubMed, Scopus, The Cochrane Central Register of Controlled Trials [CENTRAL], Science Direct, Web of Science and Google scholar) was carried out without any restrictions on date of publication to identify the reliability of saliva as a diagnostic specimen for detection of SARS-CoV-2 in suspected patients.

**Results:**

Nine eligible articles were included in this review based on our described method. All the included studies are based on clinical surveys among patients with confirmed SARS-CoV-2 infection. Most of studies included in this review, reported that there is no statistically significant difference between nasopharyngeal or sputum specimens and saliva samples regarding viral load.

**Conclusions:**

Despite limitations of this study, the findings of this review suggest that the use of self-collected saliva as a non-invasive specimen has proper accuracy and reliability regarding detection of SARS-CoV-2 based on RT-PCR technique.

## Background

During December 2019, a SARS-CoV-like coronavirus, the 2019-novel-coronavirus (2019-nCoV) was recognized in a cluster of patients with community acquired pneumonia in Wuhan, Hubei Province, China [1]. The outbreak was confirmed to be caused by a new coronavirus infection on January 10, 2020, which was named severe acute respiratory syndrome coronavirus 2 (SARS-CoV-2) by the International Committee on Taxonomy of Viruses (ICTV) [2]. And it belongs to *Betacoronavirus* genus lineage B [3].

Previous studies showed that the SARS-CoV-2 can be efficiently transmitted between people. In this regard cases of familial clustering have been documented [3]. As of May 4, 2020, more than 3 million cases of COVID-19 and 257 000 deaths have been confirmed in the world [4].

Referring to current emergency situation, preparing accurate and fast diagnostic testing methods of SARS-CoV-2 is very important with the aim of controlling the outbreak in the community and in hospitals [5]. At the time of writing this paper, PCR-based nucleic acid detection is the most effective method to diagnose suspected patients [6]. Viral pneumonias typically do not result in the production of purulent sputum, thus oropharyngeal and nasopharyngeal swabs are the recommended upper respiratory tract specimen types for SARS-CoV-2 diagnostic testing [7]. However, the collection of these specimen types requires close contact between healthcare workers and patients, which increase biosafety risk to healthcare workers through the creation of aerosol droplets. Moreover, collecting specimens with oropharyngeal or nasopharyngeal swabs may cause some degree of discomfort for patients. These methods can also cause bleeding in the target tissue especially in thrombocytopenic individuals [3].

Based on these issues, finding a safe alternative method is crucial. One of the non-invasive methods for collecting the specimens is asking patients to spit into a sterile bottle [8]. It should be mentioned that, self-collected saliva specimens in comparison with nasopharyngeal swabs can greatly decrease the chance of exposing healthcare workers to SARS-CoV-2 [9]. It has been documented that the use of human body glandular secretions, particularly saliva, as diagnostic specimens provides us with an opportunity for simpler and more efficient tool for diagnosis of viruses, especially during the critical episodes of viral diseases outbreak [10].

Previous studies showed that saliva has a high concordance rate of > 90% with nasopharyngeal specimens in the detection of respiratory viruses, including coronaviruses [11, 12]. It is noteworthy that in some cases, the researchers could have detected coronavirus just in saliva specimen rather than nasopharyngeal aspirate [11]. In this regard, high validity of diagnosing tests based on saliva specimens for SARS-CoV infections is documented [13]. In a recent animal study, the authors reported the consistent detection of SARS-CoV-2 in saliva specimens of ferrets based on quantitative real-time reverse transcription polymerase chain reaction (RT-PCR) technique [14]. The aim of this review is evaluating the available evidence regarding the efficacy of saliva as a diagnostic specimen in COVID-19 patients.

## Methods

This review was conducted following the Preferred Reporting Items for Systematic Reviews and Meta-Analyses (PRISMA) recommendations for transparent reporting of systematic reviews and meta-analyses. We did not register the review protocol because we anticipated the very limited available evidence on the topic and due to the urgency of the matter.

### Focused question

Following the PRISMA guidelines [15], a focused question was produced according to the Participants, Interventions, Control and Outcomes (PICO) principle [16]. The focused question for this review was: Is saliva a reliable diagnostic specimen for SARS-CoV-2 suspected patients compared to oropharyngeal swab tests based on RT-PCR technique?

### Eligibility criteria

Studies selected for review included original, full-text articles published in English, evaluating saliva as diagnostic specimen for detecting COVID-19 patients. All letters, narrative reviews, animal studies, and duplicate articles were excluded. The search strategy was not restricted by the publication date. Hence, all of the related evidence up to May 3, 2020, that met the inclusion criteria was assessed.

### Search strategy

A search strategy was developed to collect all scientific papers. MEDLINE (PubMed), Scopus, The Cochrane Central Register of Controlled Trials (CENTRAL), Science Direct, Web of Science and Google scholar were systematically searched up to May 3, 2020 without any restrictions on language or date of publication.

The structured search strategy used was as follows: (((saliva) OR salivary)) AND ((((((((((Novel coronavirus) OR Novel-coronavirus) OR nCoV) OR 2019 nCoV) OR 2019-nCoV) OR COVID 19) OR COVID-19) OR Wuhan coronavirus) OR Wuhan pneumonia) OR SARS-CoV-2).

Following the completion of search, the references in the papers that were selected, and also reviewed to include additional articles that were not found in the original electronic search. A number of websites that list ongoing clinical trials were also searched (http://clinicaltrials.gov, http://www.centerwatch.com/, and http://www.clinicalconnection.com). Non-scientific commentaries, reports, letters and news articles were excluded from the analysis.

### Screening of studies and data extraction

Two authors (OF and MD) independently searched through the literature. The two sets of papers were then compared. Disagreements were resolved by discussion or, if necessary, by including a third researcher (AK) to make the final decision. Duplicate articles were excluded.

One investigator (OF) extracted the data, and a second investigator (AK) checked the retrieved data independently for completeness and accuracy. The final set of selected papers and the relevant data based on our main question were summarized in Table 1.

**Table 1:** General characteristics and outcomes related to saliva specimen of the included Studies

Study team and reference	Sample	Method	Results
To KKW, et al., [9]	12 confirmed COVID-19 patients Median age: 62.5 years Age range: 37–75 years Female: 5 Male: 7	Self-collected cough out-saliva RT-PCR technique Viral culture of SARS-CoV-2 was conducted: Virus-induced cytopathic effect was examined daily for up to 7 days.	The SARS-CoV-2 was detected in saliva specimens of 11 patients (91.7%). Median viral load: 3.3 × 10^6^ copies per ml. Range of viral load: 9.9 × 10^2^–1.2 × 10^8^ copies per ml. Viral cultures were positive for three patients.
Cheng VCC, et al., [17]	One confirmed SARS-CoV-2 patient	Self-collected saliva	Viral load of the pooled nasopharyngeal and throat swab: 3.3 × 10^6^ copies per ml. Viral load of self-collected saliva: 5.9 × 10^6^ copies per ml.
Zheng S, et al., [18]	65 confirmed COVID-19 patients Median age: 65 years Male: 40 (61.5%) Female: 25 (38.5%)	Self-collected cough out-saliva RT-PCR technique	SARS-COV-2 detection rates were significantly higher in sputum (95.65%, 22/23) and saliva (88.09%, 37/42) than in throat swabs and nasal swabs (*P* < 0.001). Viral load of sputum, saliva and nasal samples were significantly higher than that of throat swabs (*P* < 0.05). No significant difference was between sputum and saliva samples regarding viral load (*P* < 0.05).
Chen L, et al., [19]	31 confirmed COVID-19 patients Median age: 60.6 years Age range: 18–86 years Female: 15 Male: 16	Saliva was collected from the opening of the salivary gland canal of cleaned oral cavity. RT-PCR technique	13 cases were tested positive for oropharyngeal swab detection. Among these 13 patients, there were 4 cases with positive detection in saliva.
To KKW, et al., [20]	23 confirmed COVID-19 patients Median age: 62 years Age range: 37–75 years Female: 10 Male: 13	Self-collected cough out-saliva RT-PCR technique	The SARS-CoV-2 was detected in saliva specimens of 20 patients (87%). The viral load in posterior oropharyngeal saliva samples was highest during the first week of symptom onset then gradually declined.
Williams E, et al., [21]	39 confirmed COVID-19 patients 50 PCR negative nasopharyngeal swabs	Self-collected saliva RT-PCR technique	The SARS-CoV-2 was detected in saliva specimens of 33/39 patients (84.6%; 95% *CI*: 70.0–93.1%) The SARS-CoV-2 was detected in 1 saliva specimen among 50 PCR negative nasopharyngeal swabs.
Zheng S, et al., [22]	96 confirmed COVID-19 patients A total of 1846 respiratory (1178 saliva and 668 sputum) samples were collected.	Self-collected cough out-saliva was collected from patients without sputum RT-PCR technique	The SARS-CoV-2 was detected in all 96 patients by testing respiratory samples.
Han MS, et al., [23]	A 27-day old neonate with COVID-19 who presented clinical symptoms	RT-PCR technique	The SARS-CoV-2 was detected in all of the neonate’s clinical specimens, including blood, urine, stool, and saliva along with the upper respiratory tract specimens.
Azzi L, et al., [24]	25 confirmed COVID-19 patients with severe or very severe disease Mean age: 61.5 years Age range: 39–85 years Female: 8 Male: 17	Self-collected saliva (drooling technique) RT-PCR technique	The SARS-CoV-2 was detected in all 25 patients’ first salivary swab In two patients the salivary samples proved positive while their respiratory swabs showed negative results on the same days.

*RT-PCR* Reverse transcription polymerase chain reaction, *SARS-CoV-2* Severe acute respiratory syndrome coronavirus 2

## Results

### Study selection

**Fig. 1:**
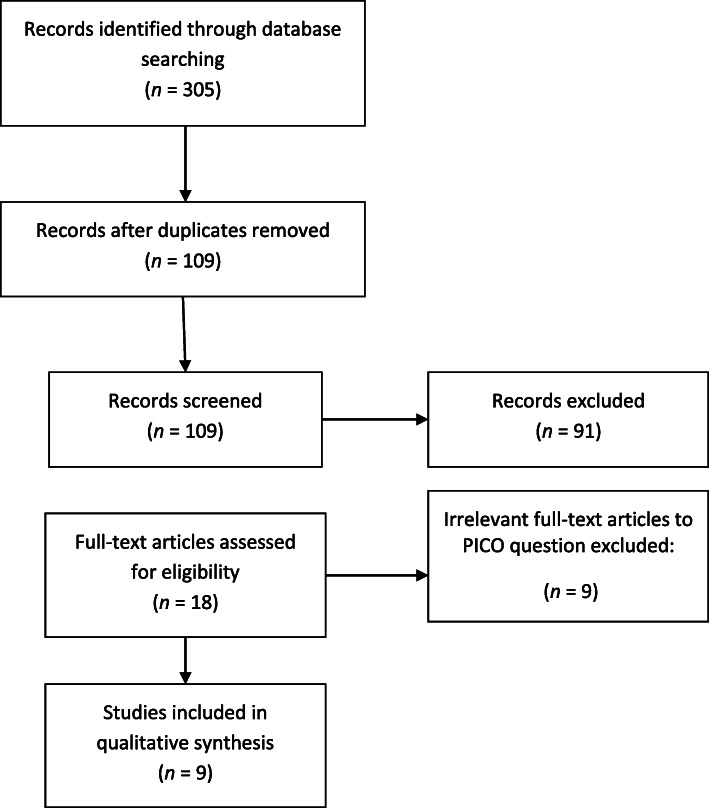
Flowchart of the process for study selection

A total of 305 publications were found as search results in six databases. By screening titles and abstracts and removing duplicates, 18 papers were retrieved, for which full text versions were obtained for detailed assessment. Manual examination of the reference lists in the 18 retrieved papers didn’t add any paper. Finally, nine eligible articles were included in the current review. More details of the data search are described in the flow chart (Fig. 1).

### Characteristics of included studies

The included studies are based on clinical surveys among patients with confirmed SARS-CoV-2 infection in China, Republic of Korea, Australia and Italy. These studies exhibited substantial heterogeneity in terms of sampling protocol, sequential of collecting samples, commercial test kit and the variety of specimens used as control group. However the laboratory method used for detection of SARS-CoV-2 are almost same in these studies. The common utilized method in this regard was reverse transcription polymerase chain reaction (RT-PCR) with some detailed differences based on the relative commercial test kit instructions. Among all, just one of these studies used viral culture technique based on saliva specimens [9].

### Laboratory results

One included studies collected the main specimens from nasopharyngeal and throat of 42 confirmed patients. However, they assessed the possibility of detection of SARS-CoV-2 from saliva specimen in just one confirmed case [17]. The results of this study showed that the viral load in saliva specimen of patient was 5.9 × 10^6^ copies per ml and 3.3 × 10^6^ in pooled nasopharyngeal and throat swab. In another study, 12 patient with laboratory-confirmed SARS-CoV-2 infection (nasopharyngeal or sputum specimens) were included [9]. The researchers reported that the SARS-CoV-2 was detected in saliva specimens of 11 patients (91.7%) in this trial. The median viral load of these 11 patients was 3.3 × 10^6^ copies per ml. It is interesting that among these SARS-CoV-2 positive cases, viral cultures were positive for three patients. Later in another article, this research team published the complementary results of their cohort study. In this paper they reported the results of investigation among 23 COVID-19 patients. The results were in accordance with the previous study and showed that the SARS-CoV-2 was detected in saliva specimens of 87% of included subjects [20].

Based on the results of included studies, three of them were performed among the Chinese participants. One of these studies included 65 cases and the other one recruited 31 confirmed COVID-19 patients [18, 19]. The results of the first project showed that the detection rate of SARS-CoV-2 based on sputum (95.65%) and saliva (88.09%) specimens were significantly higher than throat or nasal swabs (*P* < 0.001, 20). The authors also reported no significant difference between sputum and saliva samples regarding viral load (*P* < 0.05).

The study from Chen et al. showed that among the 13 patients whose oropharyngeal swab tests were positive, 4 cases were also positive for their saliva specimens [19]. The latest study among the Chinese patients, reported the results based on a total of 1846 respiratory samples (1178 saliva and 668 sputum specimens) from 96 confirmed cases [22]. The authors reported that the SARS-CoV-2 was detected in all 96 patients by testing respiratory samples [22].

The other two studies conducted in Australia and Italy among confirmed COVID-19 patients. These studies reported a detection rate of 84.6 and 100% respectively, based on saliva specimens [21, 24]. One of the included studies in this review is a case-report regarding a confirmed SARS-CoV-2 neonate [23]. In this case, the SARS-CoV-2 was detected in all of the neonate’s clinical specimens, including blood, urine, stool, and saliva along with the upper respiratory tract specimens.

## Discussion

One of the main concerns regarding epidemic prevention and control of any infectious disease is rapid and accurate screening of suspected patients. Apart from the level of sensitivity and specificity of laboratory techniques, selecting the appropriate sites to collect samples is very important. Selection of proper sampling method should be based on the tissue affinity of targeted virus, cost-effectiveness of method and also safety of patients and clinicians [18, 25]. In this study we classified the current evidence regarding the reliability of saliva as a diagnostic specimen in COVID-19 patients.

Most of the studies included in this review, reported that there is no statistically significant difference between nasopharyngeal or sputum specimens and saliva samples regarding viral load. These studies suggested saliva as a non-invasive specimen type for the diagnosis and viral load monitoring of SARS-CoV-2 [9, 17, 18, 20–22, 24]. Previous studies also reported a high overall agreement between saliva and nasopharyngeal aspirate specimens when tested by an automated multiplex molecular assay approved for point-of-care testing [12, 26, 27].

Based on these studies, the method of collection of saliva and collection device types are critical issues in the way of using saliva as diagnostic specimen. In this regard there are three main types of human saliva (whole saliva, parotid gland and minor gland) and the method of collection of each type varies accordingly [26]. When the aim of sampling is detecting the respiratory viruses with molecular assays, collecting the whole saliva from the suspected patients is useful [26]. In this regard the patients should be instructed to expectorate saliva into a sterile container. The volume of saliva should be ranged between 0.5 and 1 ml. Then 2 ml of viral transport medium (VTM) should be added to the container [11]. The next procedures will be conducted based on instructions of related RT-PCR technique in the microbiology laboratory.

The low concordance rate of saliva with nasopharyngeal specimens reported in the research of Chen et al. might be explained by the differences in the method of obtaining the samples [19]. This study reported the detection rate of SARS-CoV-2 in pure saliva fluid secreted from the opening of salivary gland canals. However in other studies patients were asked to cough out saliva from their throat into sterile containers, and hence the saliva samples were mainly sputum from the lower respiratory tract [9, 17, 18]. Thus for increasing the sensitivity of salivary tests in the way of diagnosing the suspected COVID-19 patients, the instructions should clearly explain the correct procedure to the individuals.

The use of saliva samples for diagnosis of SARS-CoV-2 has many advantages in clinical practice. First, collecting saliva is a non-invasive procedure and rather than nasal or throat swabs avoids patient discomfort. The second advantage of using saliva as specimen is related to possibility of collecting samples outside the hospitals. This sampling method doesn’t require the intervention of healthcare personnel and the suspected patients can provide it by themselves. Therefore this method can decrease the risk of nosocomial SARS-CoV-2 transmission.

Furthermore, because there is not necessary for presence of trained healthcare workers for collecting saliva specimen, the waiting time for suspected patients will be reduced. This is crucial in busy clinical settings where a large number of individuals require screening.

The results of viral culture in one of the included studies showed that saliva collected from COVID-19 patients, may contain live viruses which may allow transmission of virus from person to person [9]. These finding reinforce the use of barrier-protection equipment as a control measure, for all healthcare workers in the clinic/hospital settings during the epidemic period of COVID-19.

It should be mentioned that this study has several limitations. Firstly, the outbreak and detection of SARS-CoV-2 has begun very recently; therefore the available data in this regard is very scarce. Secondly the included studies of this review didn’t evaluate other factors such as severity of disease or disease progression that may impact on detection rate of the virus. Finally as all of the selected studies only included hospitalized confirmed COVID-19 patients, further studies should be performed in outpatient settings.

## Conclusions

In conclusion, although further research is warranted as the weight of the evidence increases, saliva can be considered as a non-invasive specimen for screening SARS-CoV-2 suspected patients. This method of sampling has proper accuracy and reliability regarding viral load monitoring of SARS-CoV-2 based on RT-PCR technique. Since oropharyngeal samples may cause discomfort to patients, saliva sampling after deep cough, could be recommended as an appropriate alternative.

## Data Availability

The dataset supporting the conclusions of this article available and will be presented based on request.

## Abbreviations

2019-nCoV: 2019-novel coronavirus
SARS-CoV-2: Severe acute respiratory syndrome coronavirus 2
ICTV: International Committee on Taxonomy of Viruses
WHO: World Health Organization
RT -PCR: Reverse transcription polymerase chain reaction
PRISMA: Preferred Reporting Items for Systematic Reviews and Meta-Analyses
SARS: Severe acute respiratory syndrome
